# Motility in blastogregarines (Apicomplexa): Native and drug-induced organisation of *Siedleckia nematoides* cytoskeletal elements

**DOI:** 10.1371/journal.pone.0179709

**Published:** 2017-06-22

**Authors:** Andrea Valigurová, Naděžda Vaškovicová, Andrei Diakin, Gita G. Paskerova, Timur G. Simdyanov, Magdaléna Kováčiková

**Affiliations:** 1 Department of Botany and Zoology, Faculty of Science, Masaryk University, Kotlářská 2, Brno, Czech Republic; 2 Institute of Scientific Instruments of the CAS, v. v. i., Královopolská 147, Brno, Czech Republic; 3 Department of Invertebrate Zoology, Faculty of Biology, Saint-Petersburg State University, Universitetskaya emb. 7/9, St. Petersburg, Russian Federation; 4 Department of Invertebrate Zoology, Faculty of Biology, Lomonosov Moscow State University, Leninskiye Gory 1–12, Moscow, Russian Federation; Institut national de la santé et de la recherche médicale—Institut Cochin, FRANCE

## Abstract

Recent studies on motility of Apicomplexa concur with the so-called glideosome concept applied for apicomplexan zoites, describing a unique mechanism of substrate-dependent gliding motility facilitated by a conserved form of actomyosin motor and subpellicular microtubules. In contrast, the gregarines and blastogregarines exhibit different modes and mechanisms of motility, correlating with diverse modifications of their cortex. This study focuses on the motility and cytoskeleton of the blastogregarine *Siedleckia nematoides* Caullery et Mesnil, 1898 parasitising the polychaete *Scoloplos* cf. *armiger* (Müller, 1776). The blastogregarine moves independently on a solid substrate without any signs of gliding motility; the motility in a liquid environment (in both the attached and detached forms) rather resembles a sequence of pendular, twisting, undulation, and sometimes spasmodic movements. Despite the presence of key glideosome components such as pellicle consisting of the plasma membrane and the inner membrane complex, actin, myosin, subpellicular microtubules, micronemes and glycocalyx layer, the motility mechanism of *S*. *nematoides* differs from the glideosome machinery. Nevertheless, experimental assays using cytoskeletal probes proved that the polymerised forms of actin and tubulin play an essential role in the *S*. *nematoides* movement. Similar to *Selenidium* archigregarines, the subpellicular microtubules organised in several layers seem to be the leading motor structures in blastogregarine motility. The majority of the detected actin was stabilised in a polymerised form and appeared to be located beneath the inner membrane complex. The experimental data suggest the subpellicular microtubules to be associated with filamentous structures (= cross-linking protein complexes), presumably of actin nature.

## Introduction

Apicomplexans (Apicomplexa Levine 1980, emend. Adl et al. 2012 [1]) belong to the most monitored group of unicellular parasites. As many of them cause major human and animal diseases, recent research has focused on the motility of apicomplexan invasive stages (zoites) representing a potential target for chemotherapeutic intervention. Apicomplexan zoites are characterised by a typical apical complex of organelles and a complicated cell cortex consisting of a continuous plasma membrane underlined by cortical alveoli (inner membrane complex = IMC). The IMC can be interrupted by micropores and connected with numerous cytoskeletal elements such as actomyosin complex, microtubules and a network of intermediate filamentous proteins [2, 3].

Although apicomplexans share a number of cytoskeletal structures with other eukaryotic organisms, a number of remarkable differences makes them unique. First of all, apicomplexan subpellicular microtubules are unusually stable and withstand high pressure, cold, and detergents that are often used for their isolation, while actin filaments (F-actin) are extraordinarily transient [2] and actin is present mostly in its globular form [4]. Except for the study demonstrating the presence of long actin filaments in *Theileria* [5], apicomplexan microfilaments can be usually observed only after treatment with F-actin stabilising drugs such as jasplakinolide [2]. So far published studies, focusing mostly on *Toxoplasma gondii* and *Plasmodium* spp., concur with the so-called glideosome concept applied for motile zoites, describing their unique mechanism of substrate-dependent gliding motility facilitated by a conserved form of actomyosin motor [6–10]. This motor is expected to be localised between the parasite plasma membrane and IMC, and the gliding is based on the locomotion of myosin along actin filaments together with the translocation of apically released adhesins to the parasite’s posterior end [11]. The above-mentioned differences in the apicomplexan cytoskeleton correspond to this machinery, which is based on and limited by the formation of transient actin filaments and their fixation to the IMC, and requires a stabile subpellicular network of microtubules.

In contrast to vertebrate pathogens, the motility mechanism in early emerging groups of Apicomplexa, such as lower coccidia and gregarines parasitising invertebrates and urochordates, still remains unclear. The basal apicomplexans studied to date are covered by a typical three-layered pellicle and use several mechanisms of motility, correlating with diverse modifications of their cortex. Among these organisms, only a few model archigregarines and eugregarines were investigated for specific aspects in their motility behaviour and related structures [3, 4, 12–43]. These studies showed that gregarine locomotion differs from the substrate-dependent gliding observed in apicomplexan zoites.

The present study focuses on the blastogregarine, *Siedleckia nematoides* Caullery et Mesnil, 1898 (Apicomplexa: Siedleckiidae), parasitising the polychaete *Scoloplos* cf. *armiger* from the family Orbiniidae. Blastogregarines are characterised by a permanent multinuclearity and complicated life cycle: gametogenesis goes through a budding of mononuclear or multinuclear spherical bodies at the posterior end of parasites and their further transformation into macrogametes and microgametes correspondingly [44]. Here, using a combined microscopic approach, for the first time we present an experimental study on the motility of the apicomplexan restricted to the marine invertebrate host.

## Materials and methods

### Material collection

The polychaetes *Scoloplos* cf. *armiger* (Müller, 1776), parasitised with *Siedleckia nematoides*, were collected at the sand-silt littoral zone at the White Sea Biological Station of M. V. Lomonosov Moscow State University (66°33.190′ N, 33°06.550′ E) and the Marine Biological Station of Saint-Petersburg State University (66°18.770’ N, 33°37.715' E), both situated in the Kandalaksha Bay of the White Sea. The polychaetes were collected within the framework of regular scientific work at White Sea Biological Station of M. V. Lomonosov Moscow State University (WSBS), which is situated in the buffer zone of Kandalaksha State nature reserve. According agreement between WSBS and the reserve, the biological station can collect animals for the scientific work on its own territory and other sites situated in the buffer zone of the reserve. The field sampling locality at the Marine Biological Station of Saint-Petersburg State University is not part of any national park or private territory, so no special permission for their collection was required. The polychaetes *S*. *armiger* are not an endangered or protected species in those regions. Animal capturing, handling and dissecting was designed to avoid distress and unnecessary suffering. Parasitological dissection of polychaetes and manipulation with parasites were performed using MBS-1 stereomicroscope (LOMO, Russia).

### Experimental motility assays and light microscopy

Parasites were treated with commercial membrane-permeable probes influencing the polymerisation of actin—jasplakinolide (JAS, Invitrogen, Czech Republic) and cytochalasin D (Invitrogen, Czech Republic), and microtubule-disrupting/antimitotic agents such as oryzalin (Sigma-Aldrich, Czech Republic) and colchicine (Sigma-Aldrich, Czech Republic). As a concentration of these probes lower than 5 μM has no obvious effect on gregarines [3], final concentrations of 10 and 30 μM for JAS, cytochalasin D and oryzalin, and 10 and 100 mM for colchicine in filtered (0.22 μm Millipore) seawater were applied to obtain reliable results on vital parasites. Cytochalasin D, JAS and oryzalin were reconstituted in dimethyl sulfoxide (DMSO) to prepare a 1 mM stock solution and diluted in filtered seawater to prepare final working concentrations, while colchicine was reconstituted directly in filtered seawater. Experimental assays that were processed for further microscopic analyses were performed in embryo dishes with a 30 mm diameter cavity. For continuous light microscopic observations of changes occurring during each assay, small pieces of host intestine with attached blastogregarines were put on single cavity microscope glass slides with a drop of drug diluted in filtered seawater. Controls were performed in filtered natural seawater and corresponding concentrations of DMSO in filtered seawater. Embryo dishes with parasites were kept in refrigerator with a temperature set point of 10°C. Behavioural and morphological changes of parasites induced by drugs’ application were monitored using a light microscope Leica DM 2000 connected to a DFC 420 digital camera. To assess the parasites’ beat frequency, the motility of *S*. *nematoides* trophozoites and gamonts attached to the host epithelium was monitored at set time intervals. The number of beats performed over period of 30 seconds was counted by taking a sample of six (at least) randomly selected individuals. Average time for each beat was calculated for the anterior-most region of the cell, where waves develop.

Three repetitions of each experiment were performed in the course of three years. Each year, for controls and every drug treatment, twelve fragments of intestines, with not less than twenty parasites attached at the experiment beginning, were investigated. During each experiment a fragment with attached parasites was periodically selected for video recording of the parasite motility under the light microscope. At the end of each experiment, these fragments were equally divided into three portions and fixed for microscopic analyses (scanning and transmission electron microscopy, confocal laser scanning microscopy).

### Electron microscopy

Specimens were fixed in an ice bath in freshly prepared 2.5–5% (v/v) glutaraldehyde either in cacodylate buffer (0.05–0.15 M; pH 7.4) or in filtered seawater. For transmission electron microscopy (TEM), the specimens were then washed 3×20 min in the same buffer as used for fixation, and post-fixed in 1–2% osmium tetroxide (OsO_4_) in the same buffer for 1–3 h. Alternatively, specimens were fixed with 3% glutaraldehyde-ruthenium red [0.15% (w/v) stock water solution] in 0.2 M cacodylate buffer (pH 7.4) and post-fixed with 1% OsO_4_-ruthenium red in the same buffer [45]. The following procedure was based on previously published protocols [46]. Observations were made using a JEM-1010 (JEOL). For scanning electron microscopy (SEM), the specimens were washed 3×15 min in the same buffer as used for fixation, processed according to Valigurová et al. [46, 47] and examined using a JSM-7401F –FE SEM (JEOL), GEMINI Zeiss Supra 40VP and REM LEO 420 (Zeiss).

### Freeze-etching

Parasitised pieces of the intestine of freshly collected polychaetes, fixed at 4°C in freshly prepared 5% (v/v) glutaraldehyde in 0.15 M cacodylate buffer (pH 7.4) or 2.5% (v/v) glutaraldehyde in 0.1 M phosphate buffered saline, were washed in the same buffer and processed according to Valigurová et al. [3] using the freeze-etching system device BAF 060 (BAL-TEC). The replicas were cleaned with 5% sodium hypochlorite, 70% sulphuric acid and 50% chromo-sulphuric acid, washed in distilled water and mounted on copper grids for examinations using a transmission electron microscope Morgagni 268 D (FEI). Evaluation of intramembranous particles (IMP) per a unit area (1 μm^2^) and the size of IMP were performed in ImageJ software. The nomenclature follows that proposed in Branton et al. [48] and used in Schrével et al. [17].

### Confocal laser scanning microscopy

Fragments of parasitised intestines were fixed for 45 min at room temperature in freshly prepared 4% paraformaldehyde in 0.1 M phosphate buffered saline (PFA) or in ice-cold methanol. Samples were carefully washed before further processing and permeabilised for 15–40 min in 0.3–0.5% Triton X-100 (Sigma-Aldrich, Czech Republic). Protocols used for the direct staining of filamentous actin with phalloidin–tetramethylrhodamine B isothiocyanate (phalloidin-TRITC; Sigma-Aldrich, Czech Republic) and indirect immunofluorescent antibody (IFA) staining using the mouse monoclonal IgG anti-actin antibody that was raised against *Dictyostelium* actin and recognises the actin in *Toxoplasma* and *Plasmodium* (provided by Prof. Dominique Soldati-Favre), mouse monoclonal anti-α-tubulin antibody (Cat. No. T5168, Sigma-Aldrich, Czech Republic), and rabbit anti-myosin antibody (Cat. No. M7648, Sigma-Aldrich, Czech Republic) follow Valigurová et al. [46, 47]. All preparations were counterstained for localisation of the cell nuclei with Hoechst 33342 (Molecular Probes, Czech Republic) and analysed using an Olympus IX80 microscope equipped with a laser-scanning FluoView 500 confocal unit (FluoView 4.3 software). Fluorescence was visualised using the TRITC (phalloidin, anti-myosin), FITC (anti-actin, anti-α-tubulin) and/or UV (Hoechst) filter sets. All specimen from one experimental assay (= particular staining and controls) were processed using the same protocol, and micrographs from confocal laser scanning microscopy (CLSM) were obtained under identical image capture conditions (filters, the laser intensity). Some micrographs were processed using the Fiji software (an image processing package based on ImageJ developed at the National Institutes of Health).

## Results

### Observations of the motility of *Siedleckia nematoides* trophozoites and gamonts

**Fig 1:**
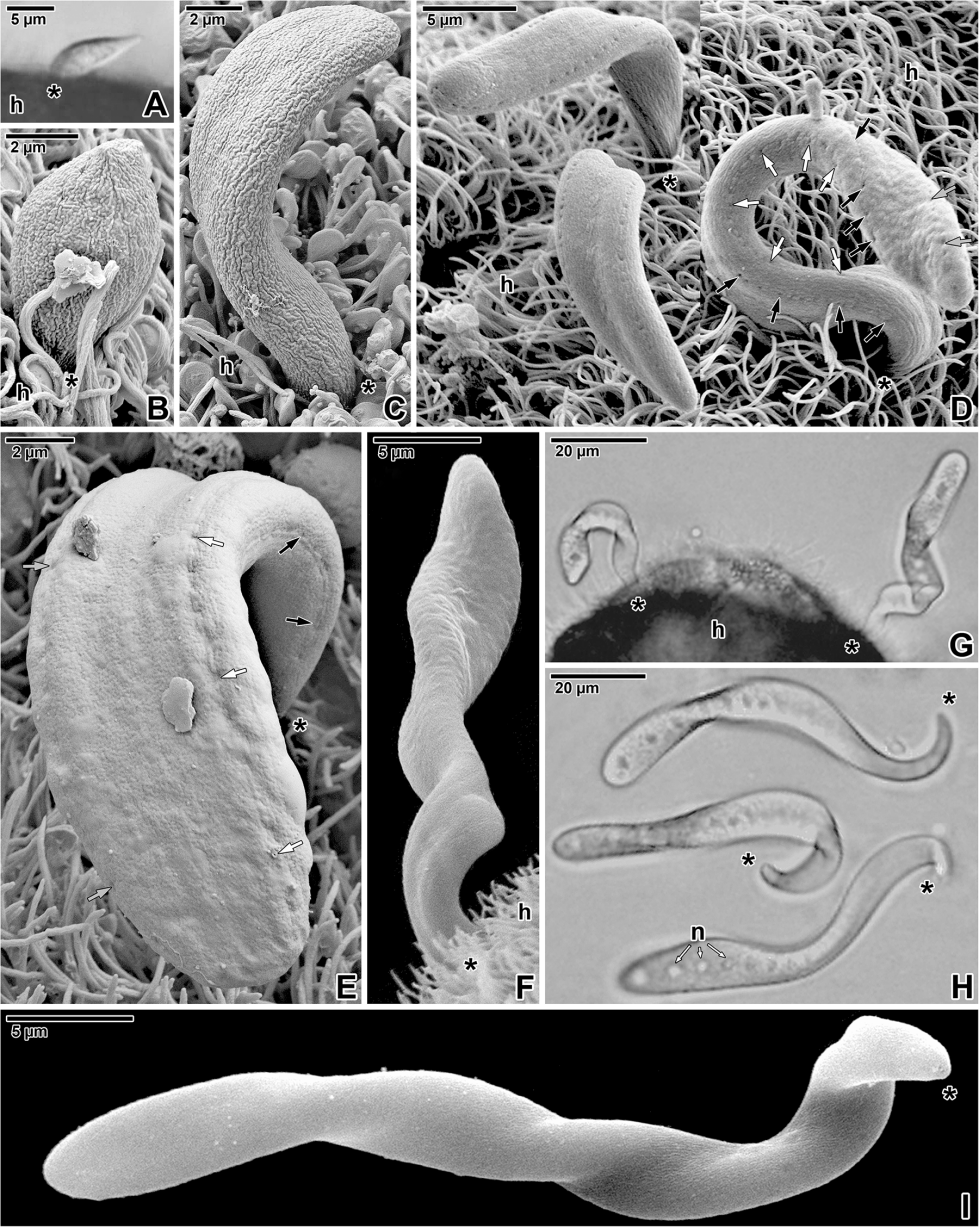
General view of *Siedleckia nematoides* trophozoites and gamonts. **A**-**B.** An early trophozoite. **C.** A young trophozoite. **D.** Composite micrograph of trophozoites attached to the host intestinal epithelium between microvilli and cilia. Note the pores organised in longitudinal rows, two per each flattened side. **E.** Attached trophozoite with a smooth surface showing the pores organised in rows. **F.** Attached gamont lacking the pores. **G.** Two parasites attached to the brush border of the host intestinal epithelium. **H.** Composite micrograph showing the sequence of movement of a single detached parasite. **I.** Detached parasite. A, G-H: LM, bright field; B-F, I: SEM. *black asterisk*–parasite apical end, *h–*host tissue, *n*–nucleus, *white/grey/black arrows*–row of pores.

Parasites developed being attached between microvilli of the host intestinal epithelium. The earliest observed stages of *S*. *nematoides* were the lancet-shaped early trophozoites (Fig 1A and 1B) exhibiting only barely visible pendular movement. The pendular movement of more advanced stage, young trophozoites (Fig 1C), was more evident. The elongated and flattened maturing trophozoites and gamonts (Fig 1D–1G) of *S*. *nematoides* exhibited very active types of movement. In addition to the pendular and twisting motility of attached parasites, their movement was typically wavy, with waves developing in the proximal region of the cell (just behind the attachment area) and proceeding to its distal end, while the last third of the cell appeared more rigid with limited mobility (S1 Video). Detached individuals either showed the same kind of movement, or simply bent from side to side with movement initiated by the proximal region (Fig 1H and 1I; S2 Video). A spasmodic movement was often observed in physiologically stressed parasites (during experiments or prolonged observations under the light microscope).

### Ultrastructural analysis of the cortex organisation

**Fig 2:**
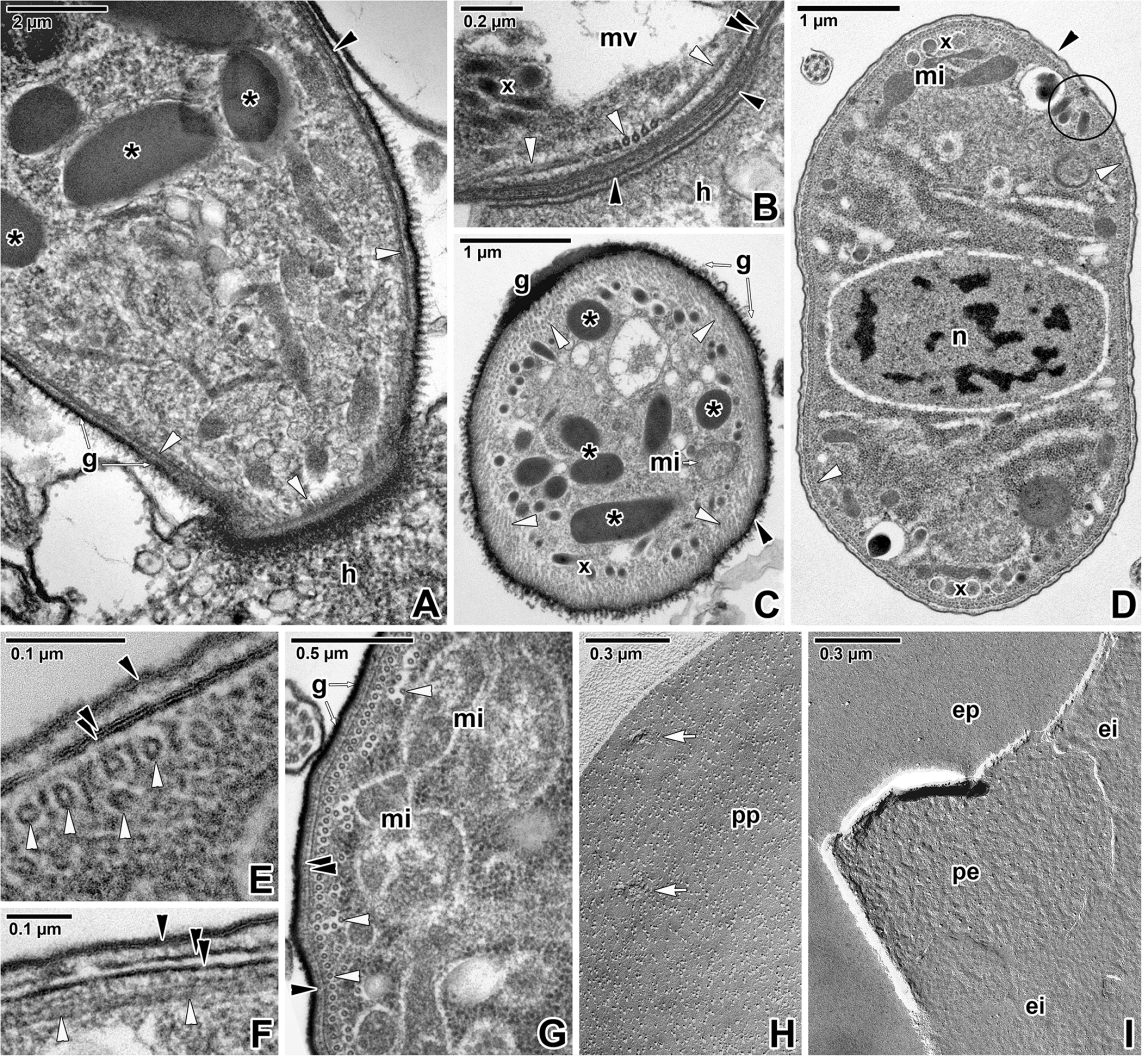
Cortex organisation in *Siedleckia nematoides*. **A.** Apical end of a parasite attached to the host enterocyte. Note the well-developed layer of glycocalyx. **B.** A detail of parasite apical end focusing on organisation of subpellicular microtubules. **C.** General view of parasite cross-sectioned in the anterior region. **D.** General view of a parasite cross-sectioned in the middle region. **E.** The cross-sectioned pellicle with well-preserved and adjacent cortical cytomembranes. **F.** Longitudinally-sectioned pellicle with obviously separated cortical cytomembranes. **G.** Cortex of parasite cross-sectioned in the middle region. **H.** Protoplasmic fracture face of the plasma membrane with pores. **I.** Fractured plasma membrane and cortical cytomembranes. A, C, G: RR TEM; B, D-F: TEM; H-I: FE TEM. *black arrowhead*–plasma membrane, *black asterisk*–rhoptry, *black circle*–pore, *double/paired black arrowhead*–IMC, *ei*–EF of the internal cytomembrane, *ep–*EF of the plasma membrane, *g*–glycocalyx, *h–*host tissue, *mi–*mitochondria, *mv*–mucronal vacuole, *n–*nucleus, *pe*–PF of the external cytomembrane, *pp*–PF of the plasma membrane, *white arrowhead*–subpellicular microtubule, *white arrows*–pores, *x—*micronemes.

Parasites attached to the enterocyte via the mucron; the cytoplasm in their anterior region contained numerous rhoptries and micronemes (Fig 2A–2C). The surface of all observed *S*. *nematoides* individuals, comprising developmental stages from early trophozoites up to gamonts, appeared smooth, lacking any grooves or folds (Figs 1B–1F, 1I, 2C and 2D).

Ruthenium red staining revealed the presence of distinct glycocalyx layer covering the entire parasite. This cell coat was evidently thicker in the parasite apical region (Fig 2A and 2C) than in its middle to distal part (Fig 2G) (apical part 85 ± 4 nm vs. distal part 26 ± 2 nm). The parasite was covered by a typical apicomplexan pellicle consisting of a plasma membrane and IMC (Fig 2E and 2F). The IMC consisted of external and internal cortical cytomembranes, from which the former was usually poorly preserved. Only a few ultrathin sections showed well preserved and closely apposed membranes of IMC (Fig 2E). More often, these two cortical cytomembranes were separated from each other, thereby forming a translucent and optically empty space between them (Fig 2F). Freeze-etching revealed that the cytomembranes were unusually undulated, while the plasma membrane was almost smooth (Fig 2H and 2I).

Analysis of the supramolecular organisation of the plasma membrane showed that intramembranous particles (IMP) are evenly distributed and arranged in a regular pattern (Fig 2H). The size of IMPs in *S*. *nematoides* pellicle membranes varied in the range from 0.5 to 21.3 nm, dependent on the membrane and its fractured face (Table 1). To compare our statistical data with known data on other apicomplexans [17, 49, 50], we additionally analysed density of IMP ranging from 6 to 14 nm (Table 2). The partition coefficient (Kp) was used as a tabulated factor for specimen comparison.

**Table 1:** The sizes and density of IMP in individual fracture faces of pellicle membranes in *Siedleckia nematoides*.

Membrane	Face	Size of IMP (nm)						Density of IMP (particles/μm^2^)
		Mean	Median	SD	SE	Min	Max	Mean ± SE
Plasma membrane	PF	7.3	7.0	2.9	0.1	0.5	21.3	3109 ± 90
	EF	4.9	4.3	2.7	0.1	0.5	19.1	314 ± 56
External cytomembrane	PF	6.8	6.6	3.1	0.1	0.5	19.1	2877 ± 213
	EF	5.7	5.2	2.4	0.1	1.0	14.1	5758 ± 357
Internal cytomembrane	PF	6.1	6.1	2.4	0.1	0.5	13.3	4352 ± 279
	EF	4.9	4.6	1.9	0.1	0.5	12.1	3411 ± 260

A total number of all sizes of IMP in membrane fracture was used for density calculation. SD–standard deviation; SE–standard error.

**Table 2:** Density of IMP (particles/μm^2^) in different apicomplexans.

Species	Plasma membrane			External cytomembrane			Internal cytomembrane		
	EF	PF	Kp	PF	EF	Kp	EF	PF	Kp
*Gregarina blaberae*^1^	977 ± 235	1469 ± 233	1.5	285 ± 39	133 ± 34	2.1	158 ± 72	297 ± 33	1.9
*Gregarina cuneata*	2770 ± 96	2244 ± 283	0.8	1420 ± 190	1260 ± 211	1.1	1502 ± 273	1993 ± 253	1.3
*Gregarina polymorpha*	2473 ± 147	1446 ± 158	0.6	602 ± 265	863 ± 202	0.7	814 ± 246	1276 ± 200	1.6
*Gregarina steini*	1783 ± 233	2265 ± 154	1.3	2588 ± 189	3820 ± 211	0.7	1886 ± 274	2339 ± 132	1.2
*Eimeria nieschulzi*^2^	218 ± 21	648 ± 73	3.0	2360 ± 133	29 ± 7	81.4	146 ± 31	1780 ± 97	12.2
*Plasmodium knowlesi*^3^	185 ± 25	2198 ± 528	11.9	1751 ± 228	38 ± 15	46.1	48 ± 28	574 ± 200	12.0
*Siedleckia nematoides*	183 ± 8	2926 ± 135	16.0	2745 ± 220	458 ± 15	6.0	797 ± 60	3342 ± 128	4.2

The size of IMP is in range 6–14 nm. Kp—partition coefficient defined as the ratio of number of particles per μm^2^ in the PF face/number of particles per μm^2^ in the EF face.

^1^Values taken from [17]

^2^Values taken from [49]

^3^Values taken from [50].

**Fig 3:**
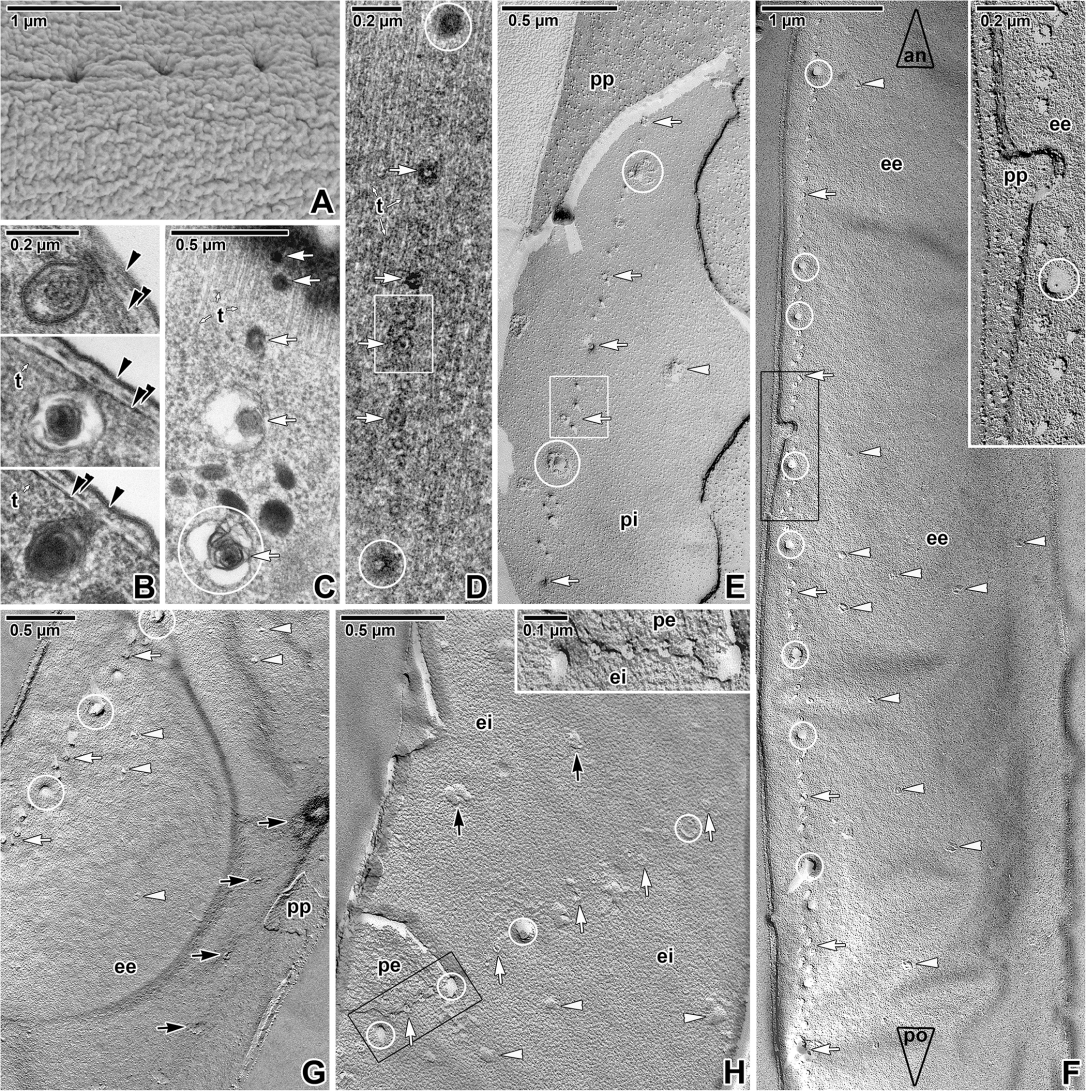
Distribution of the pores on the *Siedleckia nematoides* surface. **A.** Detail of pellicle surface with a well visible row of pores. **B.** Different longitudinally-sectioned vesicular structures connected to the pellicle and corresponding to the pores observed by SEM. **C.** An almost superficial section of a parasite revealing the pores and vesicles organised in row. **D.** Superficially-sectioned cortex showing the layer of subpellicular microtubules and a row of pores of various size. **E.** Fractured pellicle revealing the row of differently sized pores located on the PF of the internal cytomembrane, but not visible at the plasma membrane. **F.** A general view of the longitudinally fractured pellicle revealing the external cytomembrane with a lateral row of pores and few randomly distributed pores. The large empty arrowheads with labels show the direction towards anterior (an) and posterior (po) parasite ends. The inset shows the fractured pellicle and pores demarcated by black rectangle in more detail. **G.** Fractured pellicle showing pores organised in rows; few pores are distributed randomly. **H.** A fragment of fractured pellicle where several rows of variously sized pores are visible. Inset shows a more detailed view of area demarcated by black rectangle, with alternating small and large pores organised in row. A: SEM; B, D: TEM; C: RR TEM; E-H: FE TEM. *black arrowhead*–plasma membrane, *black arrows–*additional row of pores, *double/paired black arrowhead*–IMC, *ee*–EF of the external cytomembrane, *ei*–EF of the internal cytomembrane, *pe*–PF of the external cytomembrane, *pi*–PF of the internal cytomembrane, *pp*–PF of the plasma membrane, *t–*subpellicular microtubules, *white arrows*–lateral row of pores, *white arrowheads*–randomly distributed pores, *white circles* indicate some of the large pores, *white rectangle* demarcates the doubled row of pores.

Numerous pores, mostly organised in four lateral rows (two per each flattened side) running parallel to the longitudinal cell axis, were observed (Fig 1D–1E). The distance between two lateral rows was 2.77 ± 0.05 μm (= width of the cell per flattened side) and the distance between individual pores in a row was in the range from 0.5 to 1.9 μm (0.94 ± 0.06 μm). While the rows of pores were conspicuous in some specimens (Figs 1D and 3A), in others they were less distinct (Fig 1E), or even not detected (Fig 1F and 1I). Young trophozoites did not exhibit any pores at their surface under SEM (Fig 1B and 1C), but because of their sporadic presence in our samples, it remains unclear whether the presence of pores is exclusively restricted to the older stages. At least three types of different sized pores were documented: small (16.6 ± 0.9 nm), medium (48.7 ± 3.2 nm), and large (123.0 ± 4.6 nm). The small and medium pores occurred only in the fracture plane of both the cortical cytomembranes, but were never detected in the plasma membrane. All three types of pores were present in each lateral row (Fig 3C–3H). Their arrangement within rows was not regular, but the large pores usually alternated with several medium- and small-sized pores (Fig 3D–3H). In the anterior parasite region, the pores within a lateral row were organised in a single line (Fig 3F-top), while posteriorly, the small- and medium-sized pores started to form double lines (Fig 3E, 3F-bottom and 3G and 3H). The large pores were usually connected to a vesicle, containing a coiled lamellar structure or dense material and with a duct opening towards the IMC (Fig 3B and 3C). These vesicles were seen below the subpellicular microtubules, while the duct was situated in the plane of microtubule outermost layer (Fig 3B). The simple dense circle, visible in superficial ultrathin sections showing the outermost layer of subpellicular microtubules, seems to correspond to the vesicle duct, while the rosette-like organisation of dense particles could be a protein bridge connecting the vesicle duct with the pore at the IMC (Fig 3D). In replicas revealing the fractured IMC, the large pores appeared widely opened (Fig 3E). Interestingly, even in areas of lateral rows, the signs of pores were only rarely observed in fracture faces of the plasma membrane (Figs 2H and 3E). Besides the pores organised in four lateral rows, additional rows and randomly distributed pores were observed (Fig 3E–3H).

**Fig 4:**
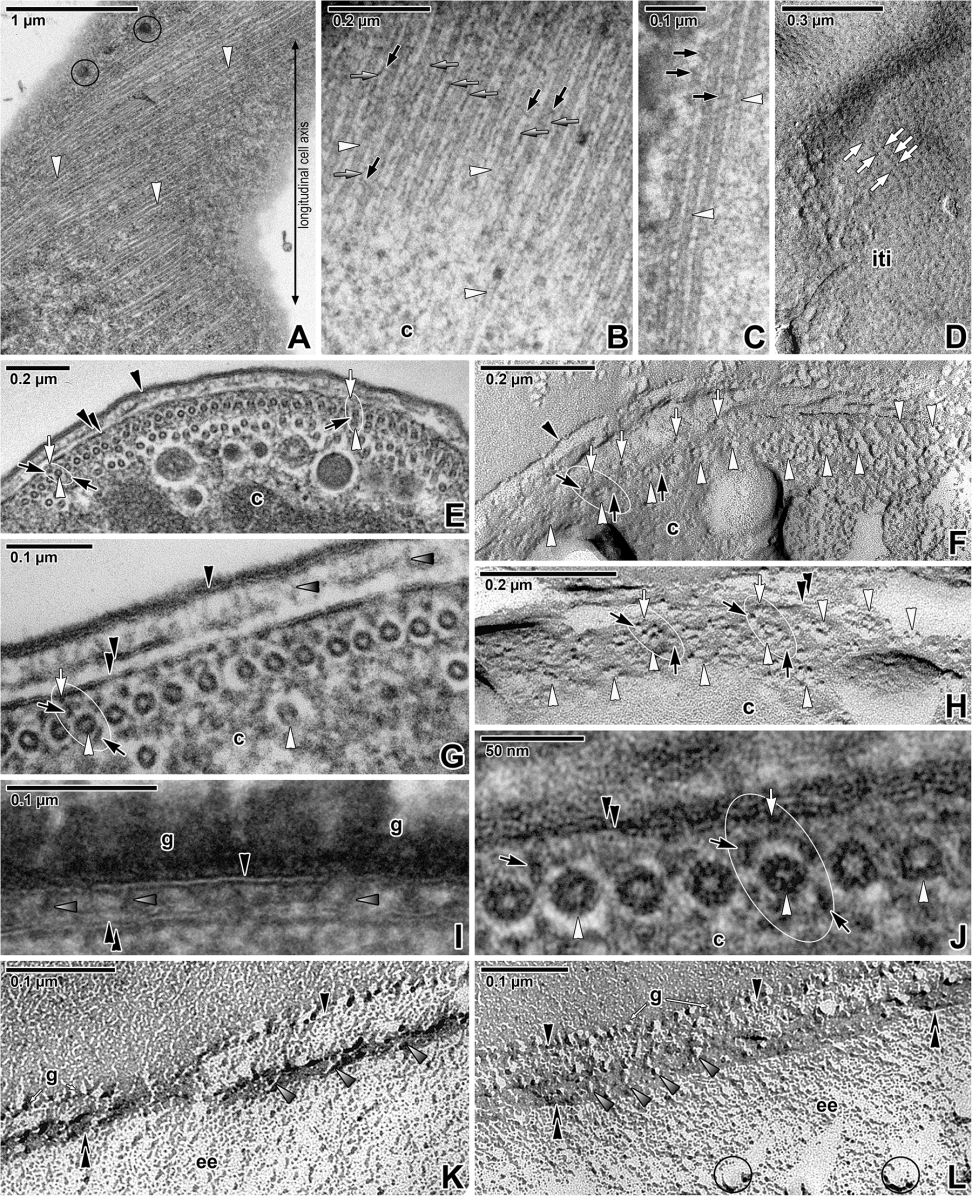
Organisation of the subpellicular microtubules in *Siedleckia nematoides*. **A.** A superficial section of a cortex revealing the pores and subpellicular microtubules being helically twisted along the longitudinal cell axis. **B-C.** Higher magnification of the longitudinally-sectioned subpellicular microtubules. Note the rows of filamentous structures running parallel to the adjacent microtubules (grey arrows) and filamentous connections with the microtubules (black arrows). **D.** Cytoplasmic face of the internal cytomembrane with IMP alignments (white arrows) that correspond to the localisation of subpellicular microtubules. **E.** The pellicle covering the anterior part of parasite, underlain by one continuous and several intermittent layers of subpellicular microtubules sectioned in cross (left) and tangential (right) plane. **F.** The view (similar to E) of fractured pellicle underlain with several layers of subpellicular microtubules. **G.** The cross-sectioned cortex in the middle region of parasite, showing the organisation of subpellicular microtubules with cross-linking protein complexes. **H.** Fractured subpellicular microtubules with cross-linking protein complexes. **I.** The detail of pellicle covered by a thick glycocalyx layer. **J.** The high magnification of cross-sectioned microtubules partially revealing the organisation of tubulin protofilaments. **K-L.** Various views of fractured pellicle revealing the cross-linking protein complexes. A, E, G, J: TEM; B-C, I: RR TEM; D, F, H, K-L: FE TEM. *black arrow–*filamentous structures around subpellicular microtubules, *black arrowhead*–plasma membrane, *c–*cytoplasm, *double/paired black arrowhead*–IMC, *g*–glycocalyx, *grey arrow–*filamentous structures located between individual microtubules, *grey arrowhead*–protein complexes localised between the plasma membrane and IMC, *iti*–inner surface of the true (= not fractured) internal cytomembrane, *white arrow*–protein complex embedded in the IMC, *white arrowhead*–subpellicular microtubule. *Black circles* mark some of the large pores. *White ellipse* encircles the cross-linking protein complexes anchoring the subpellicular microtubules to the internal cytomembrane.

The subpellicular microtubules arose from the apical pole (Fig 2A and 2B) and run to the parasite posterior end. Their arrangement appeared to be slightly helically twisted along the longitudinal cell axis (Fig 4A). Cross-sections showed the organisation of microtubules in several layers; one of them was continual and located just beneath the IMC, while the other intermittent layers were to be found deeper in the cytoplasm (Figs 2G and 4E–4H). The number of microtubule layers significantly increased towards the parasite anterior region (Fig 2C). In ultrathin sections, the outer diameter of the microtubules was 22.9 ± 0.3 nm and the inner diameter was 9.8 ± 0.5 nm, while in replicas, the outer diameter was 31.8 ± 0.5 nm and inner 14 ± 1 nm. In cross-fractured replicas, the microtubules appeared as rosettes (Fig 4F and 4H), and the diameter of the putative microtubule subunits corresponded to 5.8 ± 0.2 nm. Ultrathin sections showed individual microtubules to be localised within more lucent areas (in contrast to the surrounding cytoplasm), the so-called ‘chambers’, with a diameter of 35 ± 2 nm (Figs 2E, 2G, 4E and 4G). The distance between the IMC and microtubules forming the outer layer was about 23.5 ± 0.7 nm.

In longitudinal sections of the microtubule layer, tiny filamentous structures were detected between individual microtubules (Fig 4B). These structures, running parallel to the microtubules, seemed to interact periodically with them via short oblique filamentous connections (Fig 4B). More superficial sections revealed these connections as filamentous structures being wound around each microtubule (Fig 4C). Ultrathin cross-sections as well as freeze-etching data revealed a complex of two large and one small particles around each microtubule (Fig 4D–4H and 4J). The small particle (8.9 ± 0.6 nm in ultrathin sections, 8.8 ± 0.5 nm in replicas) was embedded in the IMC and most likely serves as an anchor for an underlying microtubule (Fig 4D–4G and 4J). One of the large particles (19.4 ± 1.2 nm in ultrathin sections, 15±1 nm in replicas) interconnected the small particle and microtubules, and was localised within the electron-lucent microtubule chamber. The second large particle was located diagonally to the first one, below the microtubule (Fig 4D–4H and 4J). All these observations suggest that these electron-dense particles may play role of cross-linking protein complexes that anchor the subpellicular microtubules to the cytoplasmic face of the internal cortical cytomembrane, thereby forming a series of microtubule-membrane bridges along entire length of each microtubule.

Filamentous structures, 3.2 ± 0.2 nm (max = 9.65 nm) thick in ultrathin sections and 5.6 ± 0.2 nm (max = 9.7 nm) thick in replicas, were seen connecting the plasma membrane with the IMC (Fig 4G and 4I). Analysis of the cross-fractured pellicle confirmed the presence of large particles between the plasma membrane and the external cortical cytomembrane (Fig 4L). In oblique fractured specimens, these appeared as short filaments situated in the supra-alveolar space (Fig 4K). In replicas, the glycocalyx was seen as an agglomeration of large particles located on the external surface of the plasma membrane (Fig 4K and 4L).

### Parasites’ motility and cortex organisation after treatment with cytoskeletal drugs

To monitor the role of individual elements of the putative motility motor in *S*. *nematoides*, the living parasites were treated with commercial probes influencing the de-/polymerisation of cytoskeletal proteins. To investigate the involvement of subpellicular microtubules in parasite motility, incubation of living parasites with oryzalin or colchicine (toxins causing the disruption of the microtubules) was performed. To verify the essential role of actin microfilaments, drugs with a contradictory effect, i.e. jasplakinolide (stabilises actin filaments and induces actin polymerisation) and cytochalasin D (disrupts actin filaments and inhibits actin polymerisation) were applied to living parasites. All experimental assays were performed on parasites attached to the host tissue and those that detached spontaneously in the course of each experiment. Treated individuals survived in extremely high doses of all of the used cytoskeletal probes and showed signs of motility for the next couple of hours (2 h in 10 mM colchicine, up to 1 h in 100 mM colchicine, 8 h in 10 μM oryzalin, 5–7 h in 30 μM oryzalin, 8 h in 10 μM JAS, 6 h in 30 μM JAS, 8 h in 30 μM cytochalasin D and more than 9 h in 10 μM cytochalasin D) (Table 3).

**Table 3:** The treatment of living individuals of *Siedleckia nematoides* with cytoskeletal drugs.

Changes / Time left after drug application	Drug / Concentration							
	Colchicine		Oryzalin		Jasplakinolide		Cytochalasin D	
	10 mM	100 mM	10 μM	30 μM	10 μM	30 μM	10 μM	30 μM
**Initial increase of movement speed (**^0^**compared to control)**	≤ 10 min	≤ 10 min	≤ 20 min	≤ 20 min	≥ 5 min	≥ 5 min	≥ 30 min	≥ 20 min
	*0.55 ± 0.03 beats/s **1.86 ± 0.11 s	*0.56 ± 0.01 beats/s **1.78 ± 0.04 s	*0.57 ± 0.03 beats/s **1.77 ± 0.10 s	*0.59 ± 0.09 beats/s **1.85 ± 0.33 s	*0.59 ± 0.04 beats/s **1.70 ± 0.09 s	*0.54 ± 0.04 beats/s **1.99 ± 0.12 s	*0.61 ± 0.05 beats/s **1.68 ± 0.11 s	*0.59 ± 0.05 beats/s **1.79 ± 0.13 s
**Oscillating movement**	+ ≥ 10 min	+ ≥ 10 min	+ ≥ 20 min	+ ≥ 20 min	-	-	-	-
**First documented decrease of movement speed**	≤ 20 min	≤ 15 min	≥ 60 min	≥ 45 min	≥ 60 min	≥ 30 min	≥ 60 min	≥ 120 min
	Δ	Δ	*0.40 ± 0.05 beats/s **2.53 ± 0.32 s	*0.39 ± 0.04 beats/s **2.64 ± 0.28 s	*0.41 ± 0.02 beats/s **2.51± 0.12 s	*0.49 ± 0.06 beats/s **2.42 ± 0.24 s	*0.43 ± 0.03 beats/s **2.42 ± 0.20 s	*0.47 ± 0.04 beats/s **2.33 ± 0.23 s
**Progressive decrease of movement speed**	≥ 30 min	≥ 20 min	≥ 120 min	≥ 60 min	≥ 120 min	≥ 60 min	≥ 300 min	≥ 240 min
	*0.23 ± 0.03 beats/s **4.88 ± 0.65 s	*0.21 ± 0.02 beats/s **5.15 ± 0.46 s	*0.18 ± 0.02 beats/s **5.92 ± 0.60 s	*0.22 ± 0.04 beats/s **5.29 ± 0.69 s	*0.35 ± 0.04 beats/s **3.51 ± 0.72 s	*0.35 ± 0.04 beats/s **3.38 ± 0.61 s	*0.27 ± 0.05 beats/s **5.19 ± 0.82 s	*0.22 ± 0.01 beats/s **4.75 ± 0.27 s
**Spasmodic movement (bending)**	-	-	+	+ Restricted to anterior region	+	+ Prevailing from side to side	+	+ Prevailing to one side
**Obvious cell rigidity (especially in posterior half)**	+	+	+	+	-	-	-	-
**Complete stoppage of motility**	≤ 120 min	≤ 60 min	≤ 480 min	≤ 420 min	≤ 480 min	≤ 360 min	≥ 540 min	≤ 480 min
**Recovery of motility in majority of blastogregarines after washing in seawater**	≤ 120 min	≤ 90 min	≥ 60 min	≥ 60 min	≥ 60 min	≥ 60 min	≥ 60 min	≥ 60 min

The symbol + indicates some observed change in the character of motility;—no obvious changes; ≥ changes appeared after the noted time period; ≤ changes appeared only during the noted time period

* beat frequency (beats/s = in Hz equivalent to 1 beat cycle per second, average ± standard error of the mean)

** beat to beat interval (an average time between the two beats ± standard error of the mean)

Δ beat frequency not measurable due to high sample variability.

^0^ Control individuals continued beating for more than 9 h (entire experiment duration); they beat at a rate of 0.51 ± 0.02 beats/s with beat to beat interval of 2.18 ± 0.13 s.

**Fig 5:**
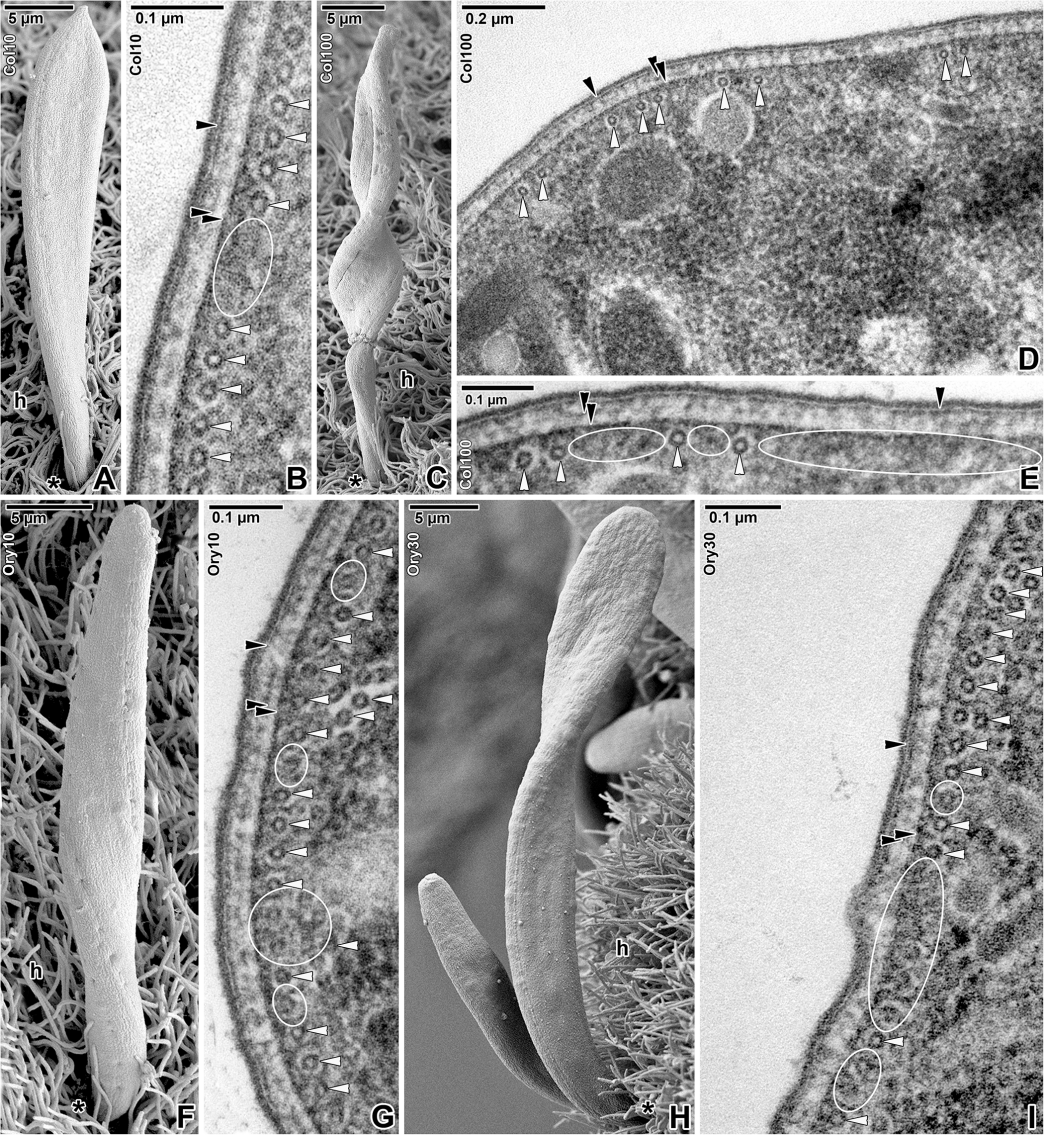
Organisation of the subpellicular microtubules in *Siedleckia nematoides* after treatment with cytoskeletal drugs. **A-B. Treatment with 10 mM colchicine for 2 h: A.** Attached gamont. **B.** Cross-sectioned cortex with subpellicular microtubules. **C-E. Treatment with 100 mM colchicine for 1 h: C.** Attached gamont. **D-E.** General view (D) and higher magnification (E) of the cross-sectioned cortex with subpellicular microtubules. **F-G. Treatment with 10 μM oryzalin for 8 h: F.** Attached gamont. **G.** Cross-sectioned cortex with subpellicular microtubules. **H-I. Treatment with 30 μM oryzalin for 7 h: H.** Attached trophozoite and gamont. **I.** Cross-sectioned cortex with subpellicular microtubules. A, C, F, H: SEM; B, D-E, G, I: TEM. *black asterisk*–parasite apical end, *black arrowhead*–plasma membrane, *double/paired black arrowhead*–IMC, *h–*host tissue, *white arrowhead*–subpellicular microtubule. *White ellipses* demarcate the regions with disrupted microtubules.

**Fig 6:**
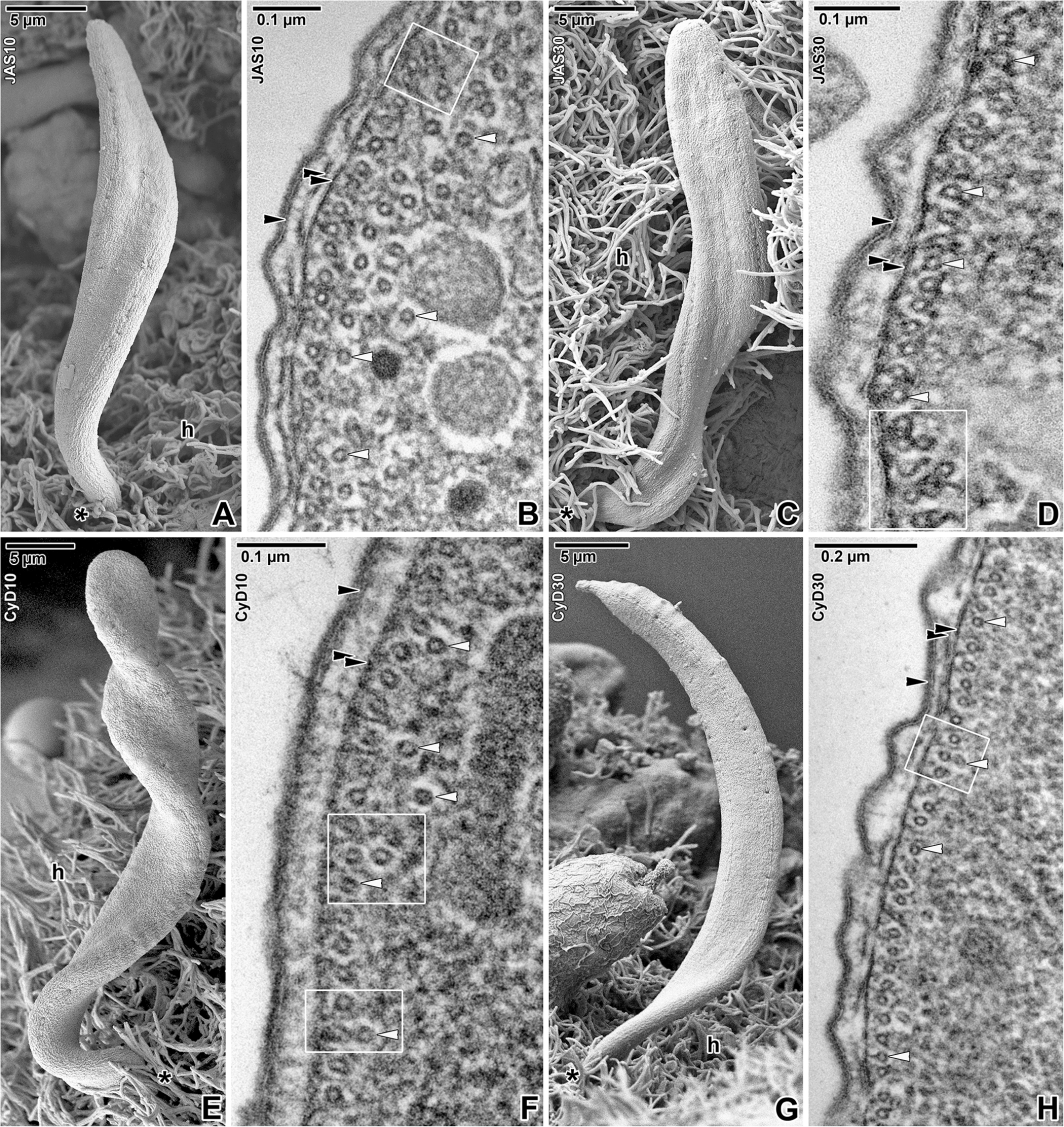
Organisation of the cortex in *Siedleckia nematoides* after treatment with cytoskeletal drugs. **A-B. Treatment with 10 μM JAS for 8 h: A.** Attached gamont. **B.** Cross-sectioned cortex with subpellicular microtubules. **C-D. Treatment with 30 μM JAS for 6 h: C.** Attached gamont. **D.** Cross-sectioned cortex with subpellicular microtubules. **E-F. Treatment with 10 μM cytochalasin D for 9 h: E.** Attached gamont. **F.** Cross-sectioned cortex with subpellicular microtubules. **G-H. Treatment with 30 μM cytochalasin D for 8 h: G.** Attached gamont. **H.** Cross-sectioned cortex with subpellicular microtubules. A, C, E, G: SEM; B, D, F, H: TEM. *black asterisk*–parasite apical end, *black arrowhead*–plasma membrane, *double/paired black arrowhead*–IMC, *h–*host tissue, *white arrowhead*–subpellicular microtubule. *White rectangles* highlight the reduced spacing between microtubule layers.

Despite extremely high concentrations of all of the applied cytoskeletal drugs, the surface of parasites appeared undamaged (Figs 5A–5I and 6A–6H) as also seen in control parasites incubated in seawater or proportionate concentrations (10 and 30 μM) of DMSO (data not shown). Furthermore, after careful rinsing and returning of the treated parasites to pure seawater, the majority recovered to normal motility in the period from 10 to 120 min (Table 3).

The treatment of parasites with antimicrotubule agents (colchicine and oryzalin) confirmed the gradual disruption of microtubules, correlating with the increasing drug concentrations and prolongation of the incubation period (Fig 5A–5I), and resulted in the complete blocking of parasite motility. During the initial acceleration in parasite movement occurring in the first 10–20 min after drugs’ application, some of the parasites showed an oscillating movement (S3 Video). Afterwards, parasites gradually decreased their movement until they completely stopped (Table 3). Drug-treated parasites exhibited irregular, spasmodic movement (mostly turning over from side to side) and the majority of them laid on the surface of the host tissue. In contrast to the very wavy movement of controls, attached parasites treated with antimicrotubule probes exhibited less pendular and twisting movements; their bodies appeared to be more rigid with obvious limitations in motility. The drug-induced cell rigidity first appeared in the posterior half of the parasite which bended in the anterior-most region (S4 Video), and afterwards the motility gradually ceased (S5 Video). During experiments with high doses of oryzalin, the frequent detachment of parasites from host tissue has been observed. The drug-treated parasites, especially those with larger dimensions (i.e. more mature), were more frequently found to be spirally curled (Fig 5C), but no signs of cell damage or collapse were seen. Ultrathin sections revealed that lower concentrations, i.e. 10 mM colchicine and 10 μM oryzalin, induced a gradual depolymerisation of subpellicular microtubules: in oryzalin treated parasites they appeared less distinct (Fig 5G), while in colchicine a few microtubules were completely lacking, as easily seen in the otherwise continuous outermost microtubule layer (Fig 5B). The majority of parasites treated with 10 μM oryzalin stopped moving after 8 h, while this effect was seen after 2 h in 10 mM colchicine. Higher doses, 100 mM colchicine and 30 μM oryzalin, obviously disrupted microtubules more rapidly, as proved by a rapid decrease of parasite motility within a considerably shorter time (20–60 min in 100 mM colchicine, 5–7 h in 30 μM oryzalin) and empty regions interrupting their outermost layer of subpellicular microtubules (Fig 5D, 5E and 5I). In comparison to oryzalin, colchicine seemed to be more efficient as it unambiguously caused the disruption of more than half of the microtubules (viewed in cross-section) in a considerably shorter time period (1 h). At the end of experiment, the number of subpellicular microtubules was 28.3 ± 1.9 per 1 μm of pellicle length in non-treated control parasites, 22.02 ± 1.9 in parasites treated in 10 mM colchicine, 11.6 ± 1.4 in 100 mM colchicine, 25.8 ± 1.3 in 10 μM oryzalin, and 20.6 ± 0.9 in 30 μM oryzalin.

When incubating with probes influencing actin polymerisation, the speed of parasite movement increased during the first 5–40 min in 10 μM JAS and 5–15 min in 30 μM JAS (S6 Video), followed by a gradual decrease of the motility intensity to zero (in 10 μM JAS after 8 h and in 30 μM JAS after 6 h) (Table 3). The movement of individuals treated with JAS appeared spasmodic and irregular, with parasites bending from side to side (S7 Video). Blocked parasites were twisted and completely non-motile. In cytochalasin D, after an initial increase of speed (30–40 min in 10 μM cytochalasin D and 20–60 min in 30 μM cytochalasin D) (S8 Video), the intensity of parasite movement gradually ceased (S9 Video) until it completely stopped in most individuals after 8 h when incubated with 30 μM cytochalasin D. Nevertheless, in 10 μM cytochalasin D, although significantly suppressed and spasmodic, the majority of parasites continued to move until the end of each experiment (more than 9 h). The ultrastructural observations in parasites after the application of actin de-/polymerising agents did not allow us to provide unequivocal interpretations (Fig 6A–6H). Ultrastructural analysis of parasites treated with JAS indicated only moderate changes in contraction and condensation of filamentous structures around the subpellicular microtubules (Fig 6B and 6D). Localisation of these structures corresponds to the cross-linking protein complexes, consisting of proteins embedded in the IMC and the network around subpellicular microtubules, which apparently anchor the microtubules to the internal cytomembrane. With an increasing concentration of JAS (10 and 30 μM), these complexes seemed to contract, thereby changing the usually regular distribution of subpellicular microtubules (Fig 6B and 6D). The most notable was the reducing or vanishing of spacing between the outer continuous and inner discontinuous microtubule layers (Fig 6B and 6D) (35.4 ± 0.4 nm in 10 μM JAS and 32.5 ± 0.2 nm in 30 μM JAS when compared to 44.8 ± 0.8 nm in controls; calculated from two adjacent microtubule layers). In individuals treated either with 10 or 30 μM cytochalasin D, density of all above-mentioned structures did not significantly differ from the normal status (Fig 6F and 6H). However, similarly to JAS, the spacing between the individual microtubule layers was reduced to 38.1 ± 1.3 nm in 10 μM cytochalasin D and 34.3 ± 1.1 nm in 30 μM cytochalasin D (Fig 6F and 6H). No conspicuous changes have been documented in the density of protein complexes localised between the plasma membrane and IMC in JAS or cytochalasin D-treated individuals (Fig 6B, 6D, 6F and 6H). The distance between the internal cytomembrane of the IMC and the outer continuous microtubule layer, however, was significantly reduced (36.3 ± 0.2 nm in controls vs. 22.5 ± 0.3 nm in 10 μM JAS, 23.7 ± 0.2 nm in 30 μM JAS, 23.0 ± 0.5 nm in 10 μM cytochalasin D, and 25.3 ± 0.3 nm in 30 μM cytochalasin D).

### Confocal laser scanning microscopic analysis of cytoskeletal structures before and after treatment with cytoskeletal drugs

**Fig 7:**
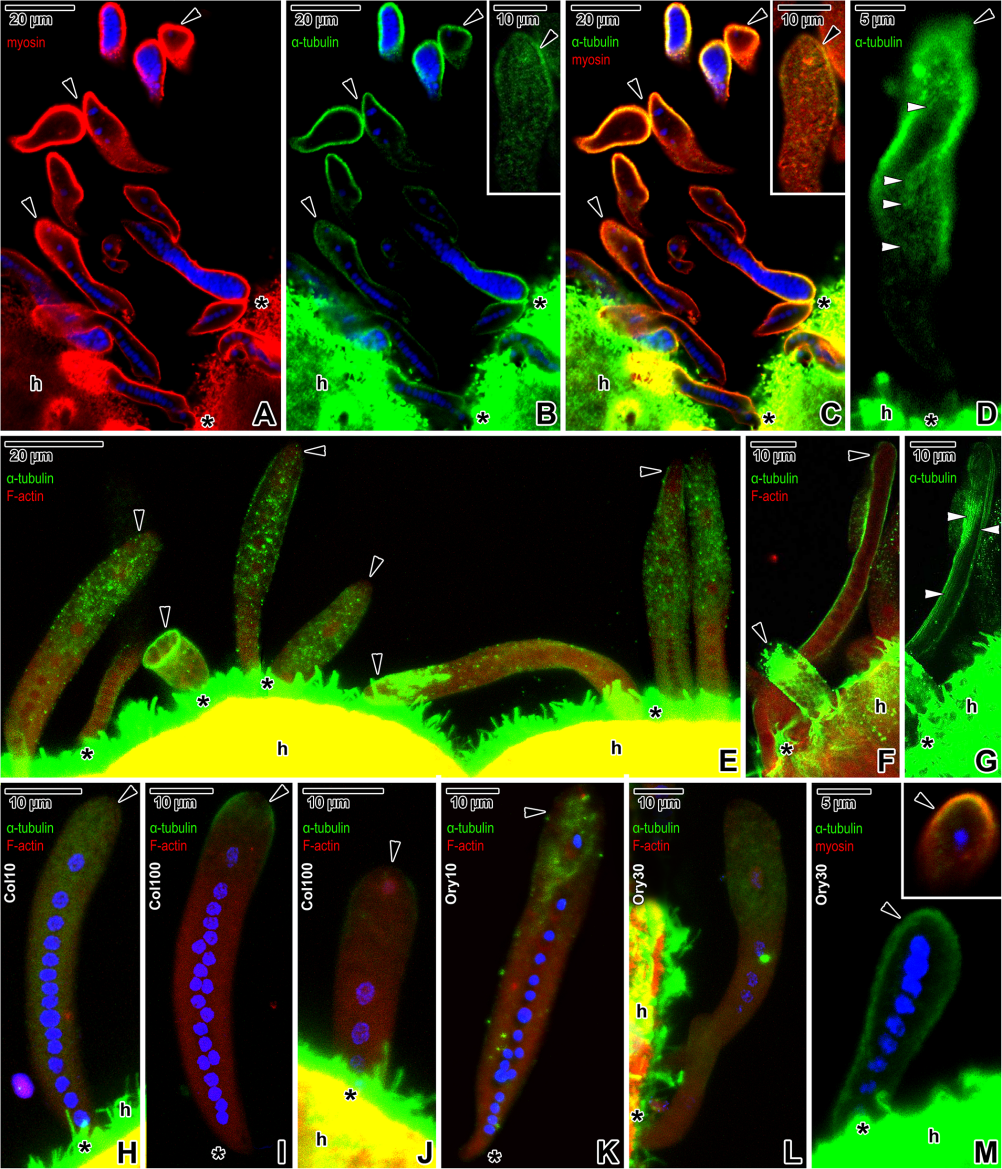
Distribution of myosin (TRITC), α-tubulin (FITC) and F-actin (TRITC) in *Siedleckia nematoides* before and after application of cytoskeletal drugs. **A–G. Non-treated parasites. A-B.** Single optical section revealing the localisation of myosin (A) and α-tubulin (B). The inset in B shows a localisation of α-tubulin in a caudal part of an individual from another optical section. **C.** Composite view showing the co-localisation of myosin and α-tubulin in a single optical section of parasites shown in A-B. The inset shows a co-localisation of myosin and α-tubulin in a caudal part of an individual from another optical section. **D.** The localisation of α-tubulin in the superficial region of a gamont. **E.** Co-localisation of α-tubulin and F-actin in parasites of various developmental stages. **F.** Composite view revealing the co-localisation of α-tubulin and F-actin in a macrogamont and microgamont in a single optical section. **G.** More superficial optical section revealing the localisation of α-tubulin in macrogamont shown in F. **H-J. Co-localisation of α-tubulin and F-actin in parasites treated with colchicine: H.** 10 mM colchicine (2 h). **I-J.** 100 mM colchicine (1 h). **K-M. Co-localisation of α-tubulin and F-actin in parasites treated with oryzalin: K.** 10 μM oryzalin (8 h). **L.** 30 μM oryzalin (7 h). **M.** Labelling of α-tubulin in a trophozoite treated with 30 μM oryzalin (5 h). The inset shows a co-localisation of α-tubulin and myosin in the trophozoite caudal region. Note the patchy distribution of α-tubulin underlying the pellicle, corresponding to the localisation of subpellicular microtubules. A-C, M: CLSM, IFA/Hoechst, methanol fixation; D: CLSM, IFA, methanol fixation; E-F: CLSM, IFA/phalloidin-TRITC, PFA fixation; G: CLSM, IFA, PFA fixation; H-L: CLSM, IFA/phalloidin-TRITC/Hoechst, PFA fixation. *black arrowhead–*parasite caudal end, *black asterisk*–parasite apical end, *h–*host tissue, *white arrowheads*–tiny longitudinal lines corresponding to the subpellicular microtubules.

Fluorescence labelling was used to visualise the arrangement of cytoskeletal structures before and after treatment of *S*. *nematoides* with cytoskeletal drugs. The myosin accumulated at the parasite periphery (Fig 7A and 7C). Similar localisation was documented in α-tubulin immunolabelling used for visualisation of the subpellicular microtubules (Fig 7B–7G). The labelling with an anti-α-tubulin antibody was also strongly positive for brush border of host intestinal epithelium. Co-localisation of myosin and α-tubulin showed both proteins continuously distributed in the cell periphery of young and mature parasites, with increasing labelling intensity towards the parasite posterior region, and overlapped to some degree (Fig 7C). The superficial optical section in the area of the parasite cortex showed patchy organisation of α-tubulin, just beneath the parasite pellicle, organised in barely visible, tiny longitudinal lines corresponding to the subpellicular microtubules (Fig 7D and 7G). Though, methanol-fixed individuals showed more intense labelling of myosin and α-tubulin (Fig 7A–7D), the less intense fluorescence signal in PFA-fixed samples allowed the pattern of cytoskeletal elements staining to be more precisely identified (Fig 7E–7G). The labelling also revealed patchy distribution of α-tubulin in the posterior half of parasites (Fig 7E). Younger parasites exhibited obviously stronger labelling of α-tubulin.

Incubation with microtubule destroying agents resulted in an overall decrease of the fluorescent signal for α-tubulin labelling (Fig 7H–7M). PFA-fixed samples were investigated for the distribution of F-actin and α-tubulin to verify that the influenced microtubules were the reason for the changes in parasite motility after the application of high doses of oryzalin or colchicine, but not potential F-actin redistribution (= treated parasites exhibited no changes in F-actin distribution; Fig 7E–7G vs. 7H–7M). Incubation with 10 mM colchicine for 2 h resulted in a more diffuse pattern of α-tubulin labelling, which was especially apparent in the caudal region of attached parasites (Fig 7H). Individuals treated for 1 h with 100 mM colchicine showed a further decrease in tubulin labelling, the localisation of which remained restricted to the caudal region only (Fig 7I and 7J). Similarly, parasites treated with 10 μM oryzalin for 8 h (Fig 7K) showed less intensive staining of α-tubulin in contrast to non-treated ones. The intensity of α-tubulin labelling in parasites treated with 30 μM oryzalin for 7 or 5 h respectively was further decreased (Fig 7L and 7M). The patchy distribution of α-tubulin organised in a line underlying the pellicle corresponded to the localisation of subpellicular microtubules (Fig 7M-inset).

**Fig 8:**
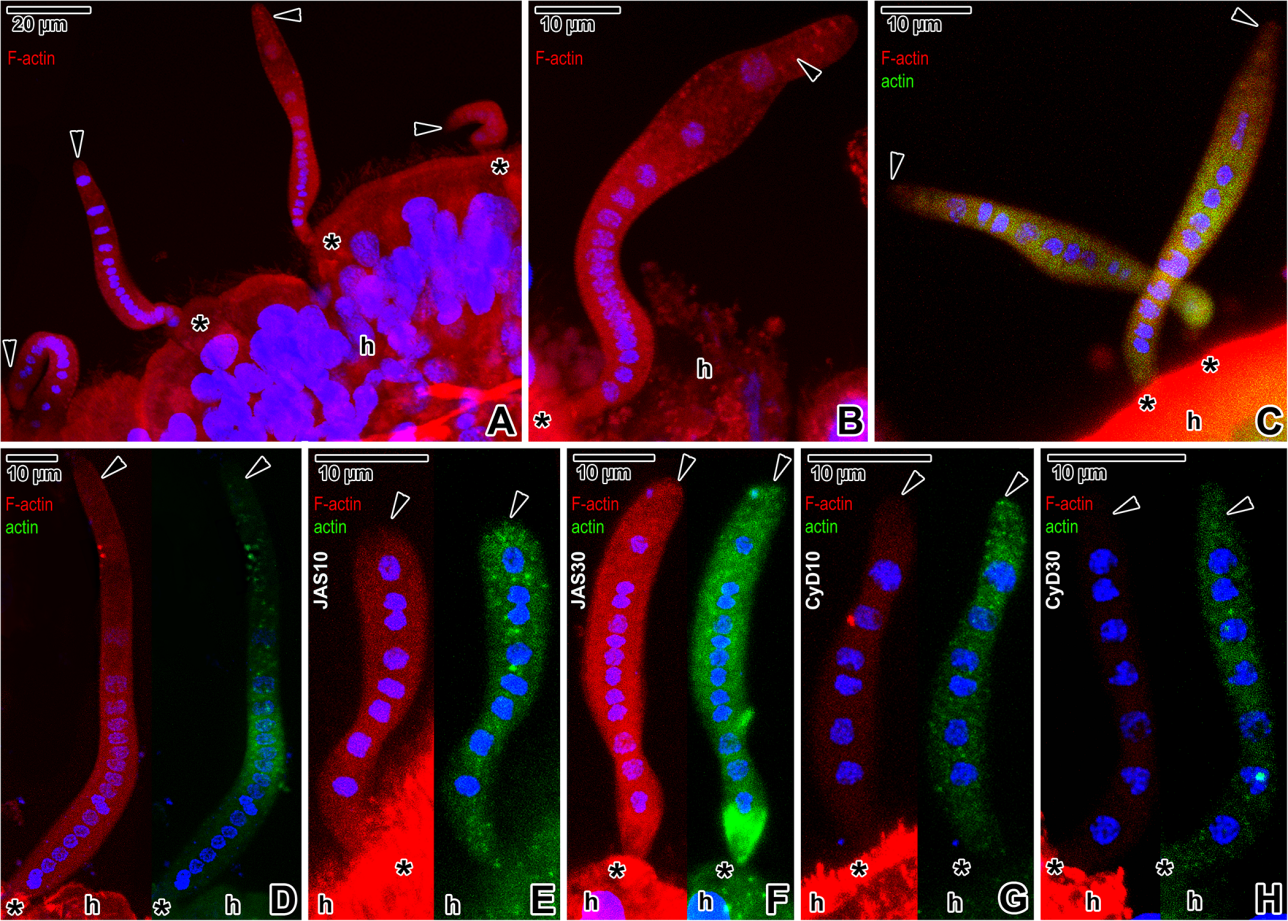
Phalloidin (TRITC) and antibody (FITC) staining of actin in *Siedleckia nematoides* before and after application of cytoskeletal drugs. **A-D. Non-treated parasites: A-B.** Localisation of F-actin with phalloidin in parasites attached to the host tissue. **C.** Double labelling with phalloidin and specific anti-actin antibody. **D.** Double labelling with phalloidin (left) and anti-actin antibody (right), image split into two separate channels. **E-F. Double labelling with phalloidin (left) and anti-actin antibody (right) in parasites treated with JAS, images split into two separate channels: E.** 10 μM JAS (8 h). **F.** 30 μM JAS (6 h). **G-H. Double labelling with phalloidin (left) and anti-actin antibody (right) in parasites treated with cytochalasin D, images split into two separate channels: G.** 10 μM cytochalasin D (9 h). **H.** 30 μM cytochalasin D (8 h). A-B, D-H left: CLSM, phalloidin-TRITC/Hoechst; C: CLSM, IFA/phalloidin-TRITC/Hoechst; D-H right: CLSM, IFA/Hoechst; A-H: PFA fixation. *black arrowhead–*parasite caudal end, *black asterisk*–parasite apical end, *h–*host tissue.

The phalloidin labelling confirmed the presence of F-actin in the blastogregarine cortex and cytoplasm (Fig 8A–8D), with a slightly increased staining in the anterior half of the cell. The pattern of staining in the caudal end exhibited a more spotted and fibrous pattern. The brush border of the host epithelium also stained intensively with phalloidin. Parasites labelled with the specific anti-actin antibody exhibited a patchy accumulation of actin (Fig 8C and 8D). Actin was distributed almost homogenously in younger stages (Fig 8C), while in mature gamonts (Fig 8D), it mostly accumulated in their middle part. The immunolocalisation of actin differed from direct F-actin labelling with phalloidin in that the antibody did not bind to the cell periphery corresponding to the cortex, and labelled the host tissue with intensity comparable to the labelling of parasites. The treatment with 10 μM JAS for 8 h resulted in the overall stronger immunolabelling of actin, with a spotted character being obvious, especially in the caudal regions of parasites (Fig 8E). The phalloidin staining of F-actin in parasites showed no or only a slight increase in fluorescence signal when compared to the control samples. The incubation with 30 μM JAS for 6 h induced a more advanced stabilisation of actin filaments, resulting in amplification of the fluorescence signal for phalloidin labelling (Fig 8F). These parasites also exhibited a strong immunolabelling of actin, distributed within entire cell and often more accumulated in parasite apical region. Parasites treated with 10 μM (9 h) or 30 μM (8 h) cytochalasin D exhibited low or almost no F-actin labelling (Fig 8G and 8H), nevertheless the signal for antibody labelling of actin did not change significantly.

## Discussion

Blastogregarines, comprising a single genus *Siedleckia*, represent rather a problematic group of unclear taxonomic position within Apicomplexa. Besides a unique life cycle [44, 51, 52], these enigmatic organisms also exhibit unusually active motility, composed of pendular and twisting movements.

### The motility in gregarines vs. the concept of glideosome in apicomplexan zoites

Typical movement generally described for apicomplexan zoites is a substrate-dependent gliding, relying on dynamic turnover of actin, the unpolymerised form of which seems to have an increased potential to form filaments relative to vertebrate actin [53]. This so-called glideosome concept, so far described for zoites of *Toxoplasma* and few other apicomplexans [6–10], requires coordinated interactions between surface adhesins and the cytoskeleton of the parasite. The actomyosin motor is described as being embedded between the plasma membrane and the IMC, and oriented by subpellicular microtubules [11]. The myosin A is linked to the IMC against the subpellicular microtubules and its head moves along the actin filament connected to a cell adhesion molecule (TgMIC2, TRAP, MIC2) [9, 10]. The complex of adhesins and actin filaments is transported towards the posterior end of the cell. As a result, the actomyosin motor generates cell gliding by transposing transmembrane adhesins through the plasma membrane by bearing against the IMC [54]. The structure and function of the glideosome require re-evaluation, as recent results have shown that numerous glideosome components (including actin and myosin) can be knocked out without complete blocking of motility [55–57].

Moreover glideosome apparently cannot be applied to all apicomplexans; e.g., gregarines exhibit diverse modes of locomotion and seem to use several mechanisms of cell motility. Different modes of gregarine motility could represent specific adaptations to a parasitism in different environments within hosts. Most eugregarines, covered by a cortex consisting of a dense array of longitudinal epicytic folds and exhibiting progressive linear gliding, were shown to use a modified machinery to move forwards on a solid surface, as they lack both the subpellicular microtubules and micronemes [3]. In contrast, coelomic eugregarines move by pulsation of their body corresponding to the peristaltic or metabolic motility accompanied with periodic changes of the body shape [24, 26–33]. The spindle shaped trophozoites and gamonts of archigregarines (*Selenidium* spp.), possessing regular sets of subpellicular microtubules, display a bending, coiling, and rolling or pendular movements along with contracting their cell shape [34–43, 58]. These movements could be also described as nematode-like [43, 58]. Both the pendular and peristaltic movement are non-progressive.

Due to similarities in external morphology and movement patterns, genus *Siedleckia* has been associated with *Selenidium* archigregarines [59]. Besides the pendular movement similar to that in *Selenidium* [34, 36, 37], additional motility modes, such as twisting, undulation, spasmodic and thrashing movements, could be observed depending on the stage and physiological status of *S*. *nematoides*. Apicomplexan zoites also show variability in their movement; e.g. circular/helical gliding (progressive) and twirling (non-progressive) of *Toxoplasma* and *Plasmodium* zoites, but their motility relies on contact with a substrate [60]. Trophozoites and gamonts of *S*. *nematoides*, however, despite bearing a striking resemblance with overgrown apicomplexan zoites, showed no signs of gliding motility. Though the pendular/twisting movements of *S*. *nematoides* might be considered to be reminiscent of twirling in *Toxoplasma* tachyzoites (occurring when the parasite rights itself vertically, remaining attached to the substrate by its posterior end and spinning clockwise) [60], the individuals of *S*. *nematoides* continued to bend/twist also after detachment from the host tissue. In this view, the movements of *S*. *nematoides* are most comparable to the attached waving of *Plasmodium* sporozoites that attach at their apical ends and then exhibit waving or flexing, or may swivel or rotate [61].

### Actin filaments

The apparent lack of visible, stable filaments does not fit for all apicomplexans, as in *S*. *nematoides*, some eugregarines and protococcidian *Eleutheroschizon duboscqi*, the phalloidin labelling revealed the presence of F-actin, even without the application of filament-stabilising probes [3, 46, 47, 62]. The research on involvement of actin- and myosin-like proteins in gregarine cell motility has been restricted to representatives of the genus *Gregarina* [3, 4, 12, 15, 16, 21]. The gregarine movement is often attributed to the F-actin cytoskeleton that is assumed to exist in the form of a myocyte (= outer layer of longitudinal and inner layer of circular myonemes) underlying the pellicle [12, 24, 25, 30, 32, 34] and to the ectoplasmic network [63], e.g. the peristalsis is accompanied by the contraction of circular myonemes [24, 25].

In S. *nematoides*, the F-actin is distributed throughout the parasite cytoplasm and cell cortex. Using a specific antibody recognising the *Toxoplasma* and *Plasmodium* actin, previous studies successfully localised the actin within both the pellicle and cytoplasm of several gregarine species [3, 46]. In *S*. *nematoides*, however, the actin immunolabelling was of patchy character and restricted to the cytoplasm only. Under TEM, only short filamentous structures with a maximal thickness of 9.7 nm were seen oriented perpendicularly between the plasma membrane and IMC. These structures did not react to the JAS or cytochalasin D treatment indicating that they are not of actin origin, and fluorescently-localised F-actin might be located deeper, i.e. beneath the IMC. Irregular distribution of microtubules observed in JAS-treated parasites (reduced spacing of individual microtubule layers occurred also in cytochalasin D-treated individuals) along with a shortened distance between the internal cytomembrane and the outer continuous microtubule layer, was probably caused by the condensation and contraction of the cross-linking protein complexes that anchor the microtubules to the internal cytomembrane. Localisation of these complexes corresponded to the filamentous structures associated with microtubules in longitudinal sections. To our best knowledge, there is no data indicating that JAS and cytochalasin D influence the polymerisation of other proteins than actin. Hence we conclude that protein complexes observed in our study could be of actin nature. Although this observation requires further analysis, it could support the idea that the actin filaments might be localised in this area. Recent studies documented the microtubule-associated F-actin in *Plasmodium* gametocytes [64]. As this actin cytoskeleton was found in non-motile gametocytes [64], it is likely that F-actin plays a rather structural role, thereby providing a template for microtubule positioning. In such cases, it must be stable rather than dynamic. Similarly to our results in *S*. *nematoides*, the treatment with cytochalasin D did not disassemble the microtubule-associated F-actin in *Plasmodium* gametocytes [64]. Another study performed on *Arabidopsis* also suggested the possibility of crosstalk between the F-actin and cortical microtubules, as JAS treatment affected the orientation and parallel ordering of microtubules and stabilised actin filaments were found to align with and move along microtubules [65]. Similarly, disruption of the actin filaments using cytochalasin B affected microtubule organisation in developing *Zinnia elegans* [66]. Altogether, these apparently highly-stabilised filamentous structures in *S*. *nematoide*s might represent novel F-actin cytoskeleton supporting the layers of numerous subpellicular microtubules.

### Subpellicular microtubules

The regularly arranged subpellicular microtubules in *S*. *nematoides* exhibit a characteristic longitudinal organisation and are nucleated from the apical polar ring, a microtubule-organising center (MTOC) unique to the Apicomplexa [2]. The helical arrangement of microtubules in apicomplexan zoites [2] and *S*. *nematoides* follow their serpentine body shape. The arrangement of subpellicular microtubules varies among Apicomplexa, but their number, length, and organisation are particular for the developmental stage of a species. The high number of *S*. *nematoides* subpellicular microtubules concurs with a positive correlation between the species dimensions and the number of subpellicular microtubules. In contrast to apicomplexan zoites with subpellicular microtubules usually ending in the region below the nucleus (2/3 of the cell length) [2], in *S*. *nematoides*, the microtubules extend along the entire length of the cell. Higher accumulation of α-tubulin in blastogregarine caudal region, accompanied by the lack of an increased number of microtubules in this area, is indicative of unpolymerised form of accumulated tubulin.

Drug-treated apicomplexans lacking subpellicular microtubules are generally non-motile and nonpolar [2]. Probes disrupting microtubules are usually effective only in certain taxonomic groups. Oryzalin is known to inhibit growth of protists but not to disrupt the vertebrate microtubules. For example, in *T*. *gondii* it binds to α-tubulin and prevents the formation of new microtubules in daughter cells, while the effect on existing stable microtubules in the mother cell is rather moderate [67, 68]; after prolonged treatment (40 h) in 2.5 μM oryzalin, all tubulin is unpolymerised and dispersed [69]. In contrast, colchicine binds to β-tubulin and effectively blocks microtubule assembly in animal cells [70]. For Chromista, corresponding to *S*. *nematoides*, the minimal effective concentration of colchicine is 10 mM [70]. Drugs disrupting dynamic microtubules are expected to be completely ineffective against the subpellicular microtubules of extracellular apicomplexans, thus indicating that these microtubules are not dynamic [68]. In *S*. *nematoides*, however, prolonged incubation in high doses of oryzalin and colchicine led to a gradual vanishing of subpellicular microtubules. The motility of drug-treated blastogregarines attached to the host tissue was often limited to their apical region, equipped with numerous layers of subpellicular microtubules, and persisted for the longest incubation period. The effect of colchicine in extremely high doses (100 mM) applied for a shorter time was considerably more effective than oryzalin. While in colchicine-treated parasites the microtubules completely disappeared in some regions, in those treated with oryzalin, they rather gradually faded away. Experiments performed on archigregarine *S*. *fallax*, using 0.1–2% colchicine diluted in seawater, showed similar results; i.e. the movement ceased in 19–360 min depending on drug concentration, while the motility of the cilia of the host's epithelium was not affected [41]. Interestingly, the pellicular folding of treated archigregarines was considerably less pronounced or non-existent, suggesting that subpellicular microtubules are important in the formation and the maintenance of the longitudinal bulges. On the other hand, the presence of the second and third row of more precisely arranged microtubules on the inner curvature of bent parasites indicates the role of microtubules in archigregarine motility [41]. Accordingly, archigregarines lacking subpellicular microtubules were shown to be non-motile, although their longitudinal bulges were supported by arrays of fibrils reminiscent of circular myonemes [39]. Our ultrastructural data on drug-treated individuals of *S*. *nematoides* suggest that continuity of the outermost layer of subpellicular microtubules is essential for normal movement. According to the doses, the motility of drug-treated individuals stopped at a specific time on a regular basis, despite obvious differences in the number of preserved microtubules in ultrathin sections. This indicates that the changes must first occur along the length of microtubules; in longitudinal sections they appeared wavy (data not shown) and, despite their presence, non-functional. Further support can be found in the observations on a differential susceptibility of *T*. *gondii* subpellicular and spindle microtubules to drugs, which could be either influenced by associated proteins specifically interacting with some of the microtubule population, or, more likely, by the length that is required to become functional [68].

The cross-linking protein complexes in *S*. *nematoides* that likely anchor the subpellicular microtubules to the cytoplasmic face of IMC, might correspond to proteins, such as microtubule-associated proteins (MAPs), which are important in controlling the local interactions of microtubules with other structures. In general, MAPs are thought to control the spacing of microtubules within the cell via microtubules interconnecting with other parts of the cytoskeleton or the plasma membrane. It is likely that heavy decoration of subpellicular microtubules in Apicomplexa may account for their unusual stability. Observations, when tubulin-specific antibodies do not label the full length of microtubules, suggest that tubulin epitopes were occluded by MAPs [71]. The MAPs, such as dyneins or kinesins, are known to be responsible for sliding between adjacent microtubules [72]. A mechanism similar to that shown in ciliary axoneme, with the microtubules sliding against one another, could account for the undulating motility of archigregarines [42, 72] and blastogregarines. MAP-based mechanism has been already proposed to explain the undulating and bending movements in *Selenidium* representatives [40–42, 72]. The sliding might be localised during the bending movements and occurred uniformly during overall contractions of a trophozoite [41]. Numerous peripheral mitochondria detected in *Selenidium* archigregarines and *S*. *nematoides* seem to play an important role in the rapid and continuous generation of ATP needed to support the highly dynamic cell plasticity ([40, 58, 72], this study), and might provide the chemical energy necessary for MAP activity [72]. Accordingly, actively moving *Selenidium* species possess more subpellicular microtubules and ectoplasmic mitochondria than less active ones. In *Selenidium*, the microtubules in deeper layers appear to be orientated obliquely to the longitudinal cell axis [58]. Although numerous ultrathin sections of *S*. *nematoides* did not allow us to unequivocally assess the orientation of deeper microtubules, in longitudinal sections they appeared to be more undulated and arranged loosely when compared to those organised in the outermost layer. In addition, single microtubules or their small clusters oriented obliquely or perpendicularly to the longitudinal cell axis were detected in few sections.

Finally, the organisation of subpellicular microtubules along with their placement within the electron-lucent hexagonal chambers (= sheaths) in *S*. *nematoides* corresponds to the cortex organisation in archigregarines of the family Selenidiidae [35, 37, 39–41, 58, 72]. The function of these chambers remains to be elucidated, but they could play an important role in microtubule sliding.

### Pellicle

In *Selenidium* trophozoites, the multi-layered pellicle, usually with broad longitudinal bulges separated by grooves [37, 58], might act as a stiff skeletal component, while the subpellicular microtubules function in cell motility [73]. It has been proposed that together they represent a unicellular analogue to the musculocuticular system of nematodes, in which longitudinal muscles function antagonistically against an elastic cuticle [41, 58]. Despite the striking similarity in motility mode and mechanism with *Selenidium*, however, the surface of *S*. *nematoides* is smooth.

The Kp of the plasma membrane in *S*. *nematoides* is significantly higher than in other apicomplexans analysed to date (Table 2), indicating that more proteins must be anchored to its protoplasmic face (the cortical supra-alveolar space). It is necessary to highlight that the widely varied sizes of IMPs in *S*. *nematoides* pellicle membranes (Table 1) had a significant impact on statistics, compared to studies, where only particles in the range from 6 to 14 nm were included in the statistical calculations ([17, 49, 50], Table 2 in this study). The IMC refers to single-membrane flattened, cortical alveoli, which underlie the plasma membrane and are coupled to a supporting cytoskeletal network of intermediate filaments [54]. Suture lines can be observed at the edges where the alveoli contact each other [74]. In the IMC of *S*. *nematoides*, no sutures were observed, so we assume that the IMC is formed from a single fused alveolus as also described in *Plasmodium* life stages (except for gametocytes) [75]. The values of Kp in cortical membranes of *S*. *nematoides* are higher than in eugregarines, but lower than in *Eimeria* or *Plasmodium* (Table 2). In apicomplexans with gliding motility, the IMC outer leaflet anchors the actomyosin motor; whereas the cytoplasmic face is intimately associated with the subpellicular microtubules and alveolins generating cell rigidity [54]. The PF of the internal cytomembrane in highly motile apicomplexan zoites shows longitudinal rows of 9 nm IMPs that likely anchor the cytoskeleton to the IMC, while the non-motile stages apparently lack them [74]. Similar subpellicular network intimately associated with the pellicle cytoplasmic face extends along the cell in *T*. *gondii* [76] and forms a resilient membrane skeleton that stabilises the alveoli [75]. Furthermore, a double linear array of IMPs might overlay the microtubules. As the helical path of some zoites during gliding corresponds to their helically coiled microtubules and linear IMPs arrays, these IMPs may function as anchorage points for an axial motor system [77]. In contrast, no regularly organised, linear IMP arrays were detected in the fractured planes of *S*. *nematoides* cortical cytomembranes. Interestingly, despite a lack of longitudinal IMP arrays in the IMC, the cytoplasmic face of the IMC exhibited imprints rather than IMP alignments matching the localisation of subpellicular microtubules (Fig 4D).

### Micropores and pores

Apicomplexan micropores are defined as organelles formed by the pellicle and composed of two concentric rings (in transverse section), the inner of which corresponds to invagination of the plasma membrane. They are generally assumed to possess a feeding function, e.g. endocytosis of the host cell cytoplasm during the vegetative phase of parasite development. In contrast to other pores, micropores are usually less numerous and widely scattered [74]. Although the presence of typical micropores (= cytostomes) has been confirmed in various apicomplexans, including extracellular gregarines [3, 18, 49, 74], no identical structures were seen in *S*. *nematoides*. Nevertheless, the pellicle of *S*. *nematoides* bears numerous pores of three different sizes; the majority of them are organised in four laterally located, longitudinal rows. Although pores were easily detected in cortical membranes by freeze-etching method, they do not seem to interrupt the plasma membrane. In *S*. *nematoides* ultrathin sections, the plasma membrane in the region of pores appears straight and not invaginated. Thus, these pores, observed only in some SEM preparations, were most likely visualised due to a specific fixative osmolarity. In eugregarines, the micropores are often located at the base of the grooves between the epicytic folds, while the smaller pores are randomly distributed on the base or on the lateral side of the folds [3, 17, 18, 78]. The diameter sizes of pores in *S*. *nematoides* correspond to those in *Gregarina* spp. [3]. In *S*. *nematoides*, the function of pores arranged in lateral rows remains to be elucidated. It is possible that they represent an alternate route to transport motor proteins between the parasite cytoplasm and the cortical supra-alveolar space, independent of the route through the apical complex, as it was described in *Plasmodium* ookinetes [74]. The largest pores in *S*. *nematoides*, connected to a vesicle containing lamellar structure, resemble the ectoplasmic structures observed in the bottom of the grooves in *Selenidium* representatives [37, 40, 58], which are assumed to serve for pinocytosis [58]. Based on comparable sizes and the putative absence of typical micropores in *S*. *nematoides*, the largest pores detected in this blastogregarine could possess similar function to the apicomplexan micropores.

### Glycocalyx

A thick cell coat, the glycocalyx, covers the cell surface of *S*. *nematoides*. The thickness of the glycocalyx layer significantly increased towards parasites’ apical end, hereby, suggesting that it is produced by some of the apical organelles; e.g. micronemes. Similar reinforcing of glycocalyx in the parasite’s apical region was documented in *Selenidium* archigregarines ([79], personal unpublished data). The often highly decorated glycocalyx of unicellular parasites allows them to interact with and respond to their environment, and is often essential for their virulence [80].

## Conclusions

Similar to archigregarines of the genus *Selenidium*, investigated blastogregarine *S*. *nematoides* infects the intestines of marine invertebrates and exhibits ecological, morphological, and motility traits inferred to reflect the early evolutionary history of apicomplexans. Despite the presence of key glideosome components such as three-layered apicomplexan pellicle, actin (including its filamentous form), myosin restricted to the cell cortex, subpellicular microtubules, numerous micronemes and prominent glycocalyx layer (where adhesins might be located), the motility mechanism of *S*. *nematoides* most likely differs from the glideosome machinery. Parasites move independently on a solid substrate and show no signs of gliding motility. We pointed to a possible role of the polymerised form of actin and tubulin in *S*. *nematoides* motility, which could be described as a combination of pendular, twisting, undulation, and sometimes spasmodic movements. Similar movements were described in *Selenidium* archigregarines. As already proposed for motility mechanism in *Selenidium* spp., our observations suggest that the subpellicular microtubules organised in several layers are the real leading motor structures. The majority of *S*. *nematoides* actin is stabilised in a polymerised form and appears to be located beneath the IMC. The filamentous structures (i.e. cross-linking protein complexes) associated with subpellicular microtubules reacted to the JAS and cytochalasin D treatment, resulting in changes in spacing of microtubules, hereby indicating that they could be of actin origin. If the axoneme-like sliding mechanism of microtubules is applicable for *S*. *nematoides* motility, it is possible that this putative actin cytoskeleton associates lengthwise with subpellicular microtubules to position them within the cytoplasm just beneath the pellicle. Otherwise, the actin filaments may force the synchronised bending of microtubules in some cell regions and this way generate the typical undulating motility of *S*. *nematoides*.

## Supporting information

S1 Video The motility of *Siedleckia nematoides* individuals attached to the host intestine during incubation in seawater. (MP4)

S2 Video The motility of detached *Siedleckia nematoides* individuals during incubation in seawater. (MP4)

S3 Video The modified motility of detached *Siedleckia nematoides* individuals incubated with 30 μM oryzalin for 20 minutes. Note: one cell demonstrates oscillating movement, while another cell–decreased bending motility.
(MP4)

S4 Video The slightly limited motility of attached and detached *Siedleckia nematoides* individuals incubated with 10 μM oryzalin for 30 minutes. (MP4)

S5 Video The limited motility of attached *Siedleckia nematoides* individuals incubated with 100 mM colchicine for 15 minutes. (MP4)

S6 Video The increased motility of attached *Siedleckia nematoides* individuals incubated with 30 μM jasplakinolide for 5 minutes. (MP4)

S7 Video The decreased motility of attached *Siedleckia nematoides* individuals incubated with 10 μM jasplakinolide for 3 hours. (MP4)

S8 Video The slightly increased and modified motility of attached and detached *Siedleckia nematoides* individuals incubated with 30 μM cytochalasin D for 25 minutes. (MP4)

S9 Video The decreased and limited motility of detached *Siedleckia nematoides* individuals incubated with 30 μM cytochalasin D for 6 hours. (MP4)

## Abbreviations

CLSM: confocal laser scanning microscopy
EF: exoplasmic fracture face
FE: freeze-etching
FITC: fluorescein isothiocyanate
IFA: indirect immunofluorescent assay
IMC: inner membrane complex
IMP(s): intramembranous particle(s)
JAS: jasplakinolide
Kp: partition coefficient
LM: light microscopy
LP: large pore
MP: medium pore
PF: protoplasmic fracture face
PFA: 4% paraformaldehyde in 0.1 M phosphate buffered saline
RR: ruthenium red
SD: standard deviation
SE: standard error
SP: small pore
SEM: scanning electron microscopy
TEM: transmission electron microscopy
TRITC: tetramethylrhodamineisothiocyanate
